# Photo-immune therapy with liposomally formulated phospholipid-conjugated indocyanine green induces specific antitumor responses with heat shock protein-70 expression in a glioblastoma model

**DOI:** 10.18632/oncotarget.26544

**Published:** 2019-01-04

**Authors:** Sayaka Shibata, Natsuki Shinozaki, Akiko Suganami, Shiro Ikegami, Yuki Kinoshita, Ryozo Hasegawa, Hirata Kentaro, Yoshiharu Okamoto, Ichio Aoki, Yutaka Tamura, Yasuo Iwadate

**Affiliations:** ^1^ National Institutes for Quantum and Radiological Science and Technology (QST), Chiba, Japan; ^2^ Department of Neurological Surgery, Graduate School of Medicine, Chiba University, Chiba, Japan; ^3^ Department of Bioinformatics, Graduate School of Medicine, Chiba University, Chiba, Japan; ^4^ Admetech Corporation, Matsuyama, Ehime, Japan; ^5^ Department of Veterinary Clinical Medicine, Faculty of Agriculture, Tottori University, Tottori, Japan

**Keywords:** glioma, PDT, photo-immune, ICG, HSP70

## Abstract

Glioblastoma (GBM) is the most common malignant brain tumor, and infiltrates into the surrounding normal brain tissue. Induction of a tumor-specific immune response is one of the best methods to obtain tumor-specific cytotoxicity. Photodynamic therapy (PDT) is known to effectively induce an antitumor immune response. We have developed a clinically translatable nanoparticle, liposomally formulated phospholipid-conjugated indocyanine green (LP-iDOPE), applicable for PDT. This nanoparticle accumulates in tumor tissues by the enhanced permeability and retention effect, and releases heat and singlet oxygen to injure cancer cells when activated by near infrared (NIR) light. We assessed the effectiveness of the LP-iDOPE system in brain using the rat 9L glioblastoma model. Treatment with LP-iDOPE and NIR irradiation resulted in significant tumor growth suppression and prolongation of survival. Histopathological examination showed induction of both apoptosis and necrosis and accumulation of CD8+ T-cells and macrophages/microglia accompanied by marked expressions of heat shock protein-70 (HSP70), which was not induced by mild hyperthermia alone at 45° C or an interleukin-2-mediated immune reaction. Notably, the efficacy was lost in immunocompromised nude rats. These results collectively show that the novel nanoparticle LP-iDOPE in combination with NIR irradiation can efficiently induce a tumor-specific immune reaction for malignant gliomas possibly by inducing HSP70 expression which is known to activate antigen-presenting cells through toll-like receptor signaling.

## INTRODUCTION

Gliomas are the most common neoplasms of the central nervous system. Further, glioblastoma (GBM) is a highly malignant neoplasm in humans; the 5-year survival rate is only 6.9%, which is lower than that for pancreatic carcinoma [1]. The standard therapy for GBM includes surgery, radiation therapy, and temozolomide combination therapy, which is characterized by a median survival time of only 15 months [2]. Thus, novel therapeutic strategies for this devastating tumor are urgently needed [3]. Since glioma cells invade normal brain tissue, tumor-specific targeting is necessary to prevent damage to the surrounding neural cells.

Photodynamic therapy (PDT), consists of a photosensitizing agent and non-ionizing excitation light, is a clinically approved and minimally invasive therapeutic procedure [4]. PDT induces heat and singlet oxygen to directly affect tumor cells, producing both necrosis and apoptosis [5]. PDT also affects vascular endothelial cells, causing shutdown of vessels and subsequent tissue ischemia, thereby accelerating cell death [6]. This treatment modality has been utilized for some cancers such as non-small cell lung cancer, esophageal cancers, and skin cancers [7]. Malignant gliomas have also been a target of PDT using Photofrin or Laserphyrin [8]. When utilized for brain tumors, it is necessary to avoid neural damage of neighboring tissues by tumor cell-specific accumulation of photosensitizers and utilization of a safe excitation light [4].

Indocyanine green (ICG) is expected to be a potent photosensitizer for PDT; however, the lack of tumor-selective retention and rapid clearance by the kidneys have limited its success [9, 10]. We have rationally designed a novel NIR photo-activating probe to be selectively trapped in the intercellular space of tumor tissue [11]. An ICG fluorophore is covalently conjugated with 1,2-dioleoyl-sn-glycero-3- phosphoethanolamine (DOPE), designated as iDOPE, for incorporation into liposome bilayers [11, 12]. LP-iDOPE is retained in tumor tissues by the enhanced permeability and retention (EPR) effect and acts as an effective photosensitizer when irradiated by NIR [4, 13]. LP-iDOPE, as with ICG, can be activated by the near infrared (NIR) light of electromagnetic spectrum from 700 nm to 2500 nm, which is characterized by a longer wavelength than the range of visible light or ultraviolet ray. ICG has absorption at around 780 nm and a high-intensity fluorescence emission at around 820 nm. Because NIR with a wavelength from 700 nm to 900 nm is not absorbed by biological tissue components such as water and hemoglobin, this range of the electromagnetic wavelength is known as the ‘biological window’ [14]. After absorption of NIR around 800nm, ICG and LP-iDOPE induce direct cellular damage including necrosis and apoptosis [14–17].

The mixture of necrosis and apoptosis can efficiently elicit an immune reaction through the accumulation of macrophages and acute inflammatory reactions [18–21] In this study, we found that one of the most important factors induced by PDT is heat-shock protein 70 (HSP70), which was reported to bind to toll-like receptor 4 (TLR4) to activate and mature antigen presenting cells such as dendritic cells (DC) [22]. We report here the experimental results of *in vivo* PDT treatment in a rat glioma model using LP-iDOPE and NIR.

## RESULTS

### Biodistribution of LP-iDOPE

**Figure 1:**
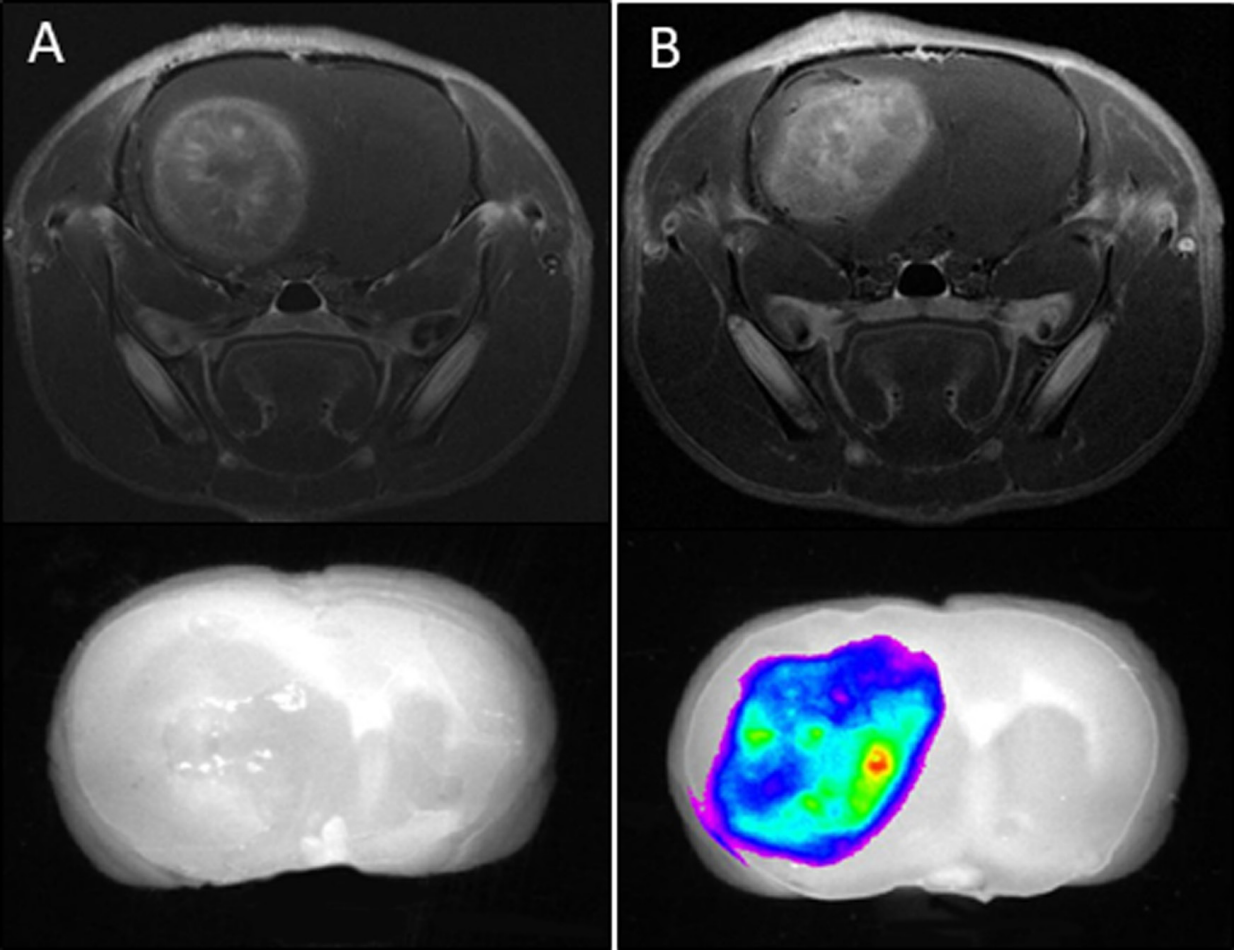
Biodistribution of liposomally formulated phospholipid-conjugated indocyanine green (LP-iDOPE) in the brain The figure shows *ex-vivo* near-infrared fluorescence images (lower panels) and *in vivo* MRI images (upper panels). (**A**) Non-treated rat, (**B**) LP-iDOPE injected rat at 24 hours after the injection. Coronal sections of the brain are shown.

To determine the distribution of LP-iDOPE in brain tumor, Gd-enhanced MRI images and NIR images were compared. Twenty-four hours after injection, rats were sacrificed and the brains removed to obtain fluorescence images with the NIR imaging system (Figure 1). Following LP-iDOPE injection, the brain tumors showed fluorescent areas consistent with the range of the Gd-enhanced areas in MRI images. In the control animals (not injected with LP-iDOPE), the intracranial tumors showed no fluorescence.

### Suppression of brain tumor growth

**Figure 2:**
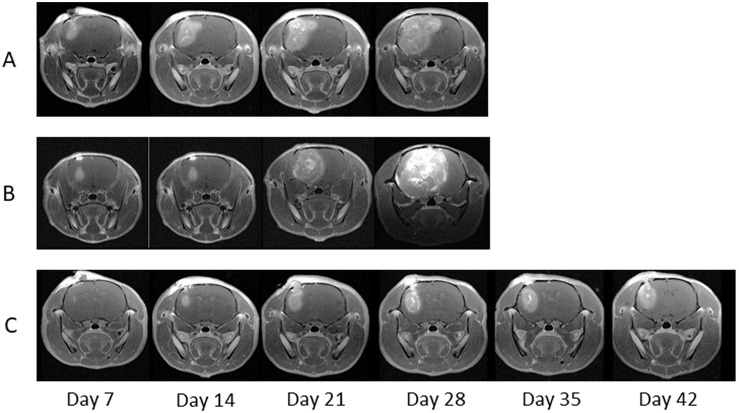
Magnetic resonance imaging (MRI) of the treated rats Serial MRIs at each week in the immunocompetent F344 rats; Group A is the non-treated control, Group B is treated with LP-iDOPE injection alone, and Group C is treated with LP-iDOPE injection and NIR irradiation.

**Figure 3:**
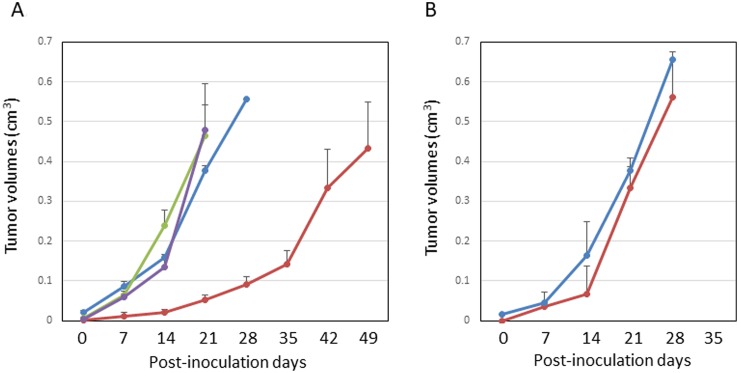
Growth suppression in the rat glioblastoma model (**A**) Line graphs of the intracerebral tumor volume measured by serial MRIs at each week in the immunocompetent F344 rats. The blue line indicates the non-treated control (Group A, *n* = 3), The green line indicates treatment with LP-iDOPE injection alone (Group B, *n* = 4), the purple line indicates treatment with hyperthermia alone at 45° C (*n* = 3), and the red line indicates treatment with LP-iDOPE injection and NIR irradiation (Group C, *n* = 6). (**B**) Line graphs of the intracerebral tumor volume measured with serial MRIs at each week in the immunodeficient rats (nude rats). The blue line is non-treated control (*n* = 1), and the red line is treated with LP-iDOPE injection and NIR irradiation (*n* = 3). The bars indicate the standard error of the mean.

**Figure 4:**
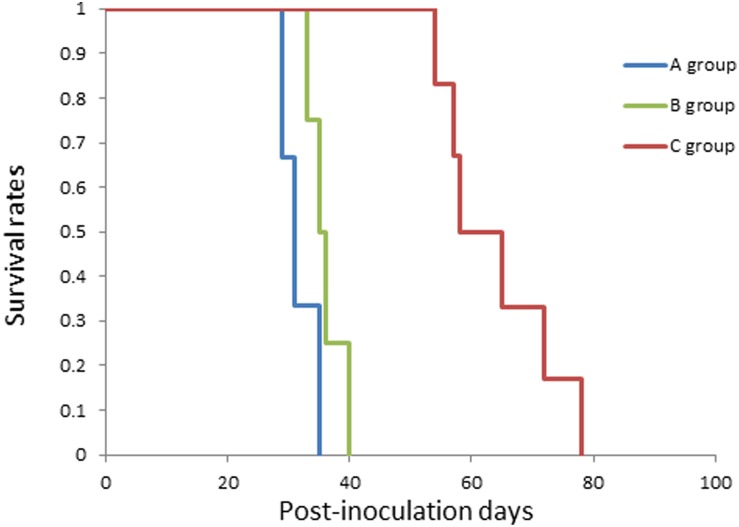
Kaplan–Meier survival curve of the treated rats The blue line indicates the non-treated control (A group, *n* = 3), the green line indicates treatment with LP-iDOPE injection alone (B group, *n* = 4), and the red line indicates treatment with LP-iDOPE injection and NIR irradiation (C group, *n* = 6).

Since 9L gliosarcoma cells are syngeneic to Fischer 344 rats, the inoculation of 9L cells into rat brains resulted in 100% tumor establishment. We generated brain-tumor-bearing rats, which were divided them into three groups: Group A (control) was not administered NIR irradiation or intravenous injection, Group B was administered LP-iDOPE injection only, and Group C was administered NIR irradiation and LP-iDOPE injection concurrently. Gd-enhanced MRI was performed weekly to evaluate tumor volume (Figure 2). The mean tumor volume of Group A increased from 0.020 ± 0.005 cm^3^ at 1 week after tumor inoculation to 0.378 ± 0.012 cm^3^ at 4 weeks. The mean tumor volume of Group B increased from 0.004 ± 0.003 cm^3^ to 0.478 ± 0.116 cm^3^, while that of Group C increased from 0.002 ± 0.002 cm^3^ to 0.053 ± 0.012 cm^3^. The tumor growth of Group C was significantly suppressed compared to the other two groups (Figure 3A) (Group C vs. Group A, *p* = 0.0339; Group C vs. Group B, *p* = 0.0209, Mann–Whitney's *U*-test). The mean survival time of each group was 31.6 ± 3.1 days for Group A, 36.0 ± 2.9 days for Group B, and 64.0 ± 9.4 days for Group C. Kaplan-Meier analysis showed that the animals in Group C survived significantly longer than the other groups (Log-rank test, Group C vs. Group A, *p* = 0.0018; Group C vs. Group B, *p* = 0.0007) (Figure 4).

### Effects in immuno-deficient rats

Immuno-deficient nude rats were used to ascertain whether the immune system contributes to the tumor suppressing effect observed in the Fischer 344 rats. The mean tumor volume of the tumors treated concurrently with LP-iDOPE injection and NIR irradiation at 4 weeks after inoculation was 0.33 ± 0.02 cm^3^, which was not different from that of the non-treated control (0.38 cm^3^, *p* = 0.362) (Figure 3B). This result suggests that the immune reaction has an important role in the effect of PDT using LP-iDOPE injection and NIR irradiation.

### Effect of heating alone

Since the PDT system increases the tissue temperature by a maximum of 10° C, we examined the effect of heating to 45° C alone. At this temperature, there was no difference between the non-treated control and the tumors treated with LP-iDOPE injection and NIR irradiation in tumor volume (Figure 3A) and histopathological findings (data not shown).

### Histopathological analysis

**Figure 5:**
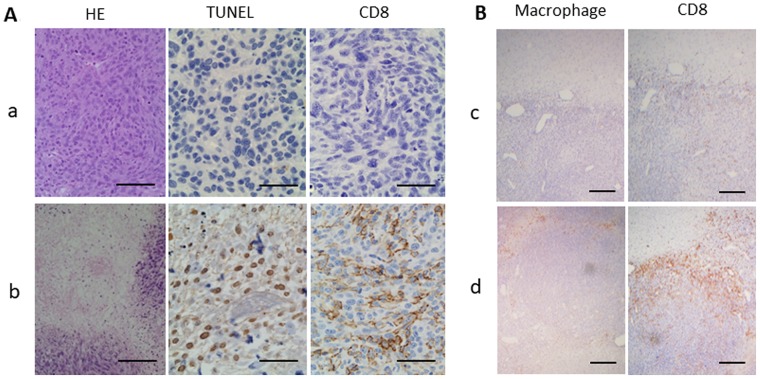
Histopathological analyses of the tumors obtained at 2 weeks after treatment initiation (**A**) Hematoxylin and eosin (HE) staining, TUNEL staining, and immunohistochemistry for the anti-CD8 antibody OX8. (a) Non-treated control, (b) treated with LP-iDOPE injection and NIR irradiation. Scale bar indicates 100 μm. (**B**) Immunohistochemistry for anti-rat macrophage/microglia (RM-4) and for CD8+ T cells. (c) mild hyperthermia at 45° C, and (d) treated with LP-iDOPE injection and NIR irradiation. Scale bar indicates 200 μm.

**Figure 6:**
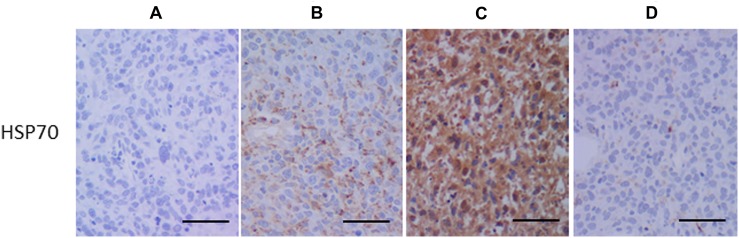
Immunohistochemistry for heat shock protein-70 (HSP70) (**A**) non-treated control, (**B**) mild hyperthermia at 45° C for 10 min, (c) treated with LP-iDOPE injection and NIR irradiation, and (d) treated with interleukin-2 cDNA-transduction. Scale bar indicates 100 μm.

Large necrotic components with islands of viable cells were observed in HE staining (Figure 5A). The TUNEL assay showed that TUNEL-positive cells with shrunken nuclei were clustered in GBM tissues, while no TUNEL-positive cells were observed in control specimens. CD8 immunostaining showed infiltration of CD8+ cells in glioma tissues treated with LP-iDOPE injection and NIR irradiation, while no CD8+ cells were found in the control specimens (Figure 5A). Although RM-4+ cells were scarcely observed in the non-treated tumors, there were some positive cells in the peripheral region of the tumors treated with PDT, in which many CD8+ cells were also located (Figure 5B). Activation of the innate immunity is thought to be necessary to effectively initiate specific acquired immunity. Therefore, we examined HSP70 expression, which is reported to be specifically induced by PDT, and confirmed that this protein was extensively expressed in the treated tumors; in contrast, HSP70 was not induced in the control, heated-only tumors, or IL-2 producing 9L tumors (Figure 6). These results indicate that LP-iDOPE injection with NIR irradiation induced diffuse cellular death, mixed with necrosis and apoptosis, in tumor tissues through a 9L-specific immune reaction induced by PDT.

## DISCUSSION

Because ICG has a strong absorption band between 700 and 800 nm, which is in the “biological window”, deep tissue penetration is expected to occur without causing significant damages to normal tissues [14–16]. However, utilization of ICG for PDT has been limited due to its rapid clearance from vessels and organs [18, 19] We showed here that LP-iDOPE accumulates in Gd-enhanced glioma tissues by MRI and the concurrent application of NIR effectively suppressed intracerebral tumor growth. The tissue temperature after LP-iDOPE injection with NIR irradiation increased up to a maximum of 10° C in our previous study [11, 12] We initially supposed that heat injury as a photothermal effect was the most important factor for tissue damage. However, direct tissue hyperthermia at 45° C for 10 min showed no effects on MRI or histological analyses. On the other hand, there were numerous CD8+ T-lymphocytes in the treated GBM tissues, while the therapeutic effects were lost in the immunocompromised animals. Therefore, it is possible that the host immune reaction to the tissues treated by PDT with LP-iDOPE was an important factors for the therapeutic efficacy.

Histopathological examination indicated that a mixture of apoptosis and necrosis was induced by PDT with LP-iDOPE, which was reported to be the characteristic form of cellular death in PDT [4, 5]. The relationship between the mode of tumor cell death and the efficiency of the immune response induction remains controversial. Recently, in the case of necrosis, cytosolic constituents have been shown to spill out into the extracellular space and provoke a significant inflammatory response, which attracts host leukocytes into the tumor tissues and increases antigen presentation [18–20] In contrast, apoptotic cell death is also important for the expression of a full repertories of antigens by antigen-presenting cells like DC because apoptotic cells can be phagocytosed by DCs [21] Thus, an optimal combination of apoptosis and necrotic cell death, in contrast to other cytotoxic agents which only trigger apoptotic cell death, may be important for the efficient induction of an anti-tumor immune response in PDT [21]. We also confirmed that HSP70, a stress-inducible chaperone protein, [22] was strongly expressed in the LP-iDOPE-treated NIR-exposed tumor tissues compared to the non-treated control or IL-2-producing tumor cells. Intracellular HSP70 binds to cellular peptides to mediate the formation of MHC class I-binding complex, thereby facilitating antigen presentation [23, 24] Extracellular HSP70 is an important endogenous ligand for TLR-4 to induce the activation and maturation of DCs, which bridge innate and acquired immunities. In addition, various immune mediators are reported to be induced by non-lethal PDT in glioblastoma cell lines. Kammerer *et al*. reported that the most prominent upregulated genes are the chemokine genes CXCL2, CXCL3, and IL-8/CXCL8 as well as IL-6, which are mainly correlated with the induction of reactive oxygen species such as singlet oxygen and can stimulate proinflammatory reactions through 5-aminolevulinic acid, which is a metabolic precursor of the photosensitizer protoporphyrin IX [25]. A similar significant induction of chemokines and cytokines was reported for ICG-mediated PDT [25] Enhancement of immune reactions by PDT using LP-iDOPE can be termed photo-immune therapy.

Our results showed that the thermal effect alone at 45° C for 10 min was not effective for inducing cell death. Previous reports showed that mild hyperthermia around 42–43° C caused apoptotic cell death depending on the exposure time [26, 27]. We speculate that moderate hyperthermia at 45° C is not effective for directly inducing cell death in glioma cells, but could potentially enhance the antitumor immune reactions collaborating with singlet oxygen in PDT. Recently, Tsang *et al*. reported that mild hyperthermia induced infiltration of leukocytes and macrophages to create a favorable microenvironment for an immunological chain reaction facilitating tumor cell death [28].

In the present study, we showed that PDT using LP-iDOPE was an effective treatment method for a rat glioblastoma model partly through activation of innate immunity. In the clinical situation, ICG has been well studied using near-infra-red spectroscopy (NIRS) to evaluate cerebral blood flow (CBF) [29] NIR light can reach as deep as 2.5–3 cm from the scalp into the cerebrum. This suggests that tumors near the brain surface could be effectively treated using this method. For deep-seated tumors, we are planning to apply intraventricular endoscopy and stereotactic insertion of a laser probe into the tumor core. After being granted Federal Drug Administration (USA) approval in 1959, ICG was used clinically as a diagnostic agent in various occasions. ICG was administered in order to confirm perforator patency following aneurysmal clipping in neurosurgery. Liposomes have also been used clinically as a carrier agent. Since LP-iDOPE was created by a combination of ICG and liposomes, its safety is sufficiently high for early clinical application. We are now planning to conduct a clinical trial of LP-iDOPE with NIR irradiation for newly-diagnosed GBM in human.

## MATERIALS AND METHODS

### Animals and brain tumor model

9L gliosarcoma cells or interleukin-2 cDNA transduced 9L cells, syngeneic to Fischer 344 rats, were prepared to a concentration of 1~5 × 10^5^ cells/μl in 2 μl of PBS [18]. These cells were injected intra-cranially using a micro-injector over 5 min into experimental animals. Male Fischer 344 rats, weighing between 200 and 240 g (7–8 weeks old), were anesthetized with 2.0% isoflurane (Abbott Japan, Tokyo, Japan) and placed on a stereotactic apparatus. A burr hole was made 1mm posterior to the bregma and 3 mm right of the midline. A 25-gauge needle was inserted at a point 3mm ventral from the dura. The rats were observed daily until severe paresis, ataxis, periophthalmic encrustations, or >20% weight loss developed. Immunocompromised nude rats were used to confirm the possible contribution of the immune system to the therapeutic effects of LP-iDOPE. These animals were maintained in a specific pathogen-free environment in accordance with the Laboratory Animal Resources Commission Standards. The animal experimentation was reviewed and approved by the Institutional Animal Care and Use Committee of the National Institute of Radiation Research.

### Preparation of LP-iDOPE

The preparation and physicochemical properties of LP-iDOPE have been described elsewhere [9]. An ICG fluorophore is covalently conjugated with 1,2-dioleoyl-sn-glycero-3-phosphoethanolamine (DOPE) for incorporation into liposome bilayers. In this study, we selected 10 mol% iDOPE to achieve a high fluorescence intensity by preventing fluorescence quenching between neighboring iDOPE species. The liposome solution was filtered through a 10 nm pore polycarbonate filter. LP-iDOPE was synthesized to an average size of 191 nm, which satisfied the restriction of the EPR effect. The peak wavelength of absorbance of LP-iDOPE ranged from 780 to 800 nm, and the peak wavelength of fluorescence ranged from 800 to 820 nm.

### LP-iDOPE injection and NIR irradiation

LP-iDOPE was injected via the tail vein of glioma-bearing rats at a concentration of 4 μM after confirmation of tumor establishments at day 7. A NIR excitation pulse beam (300 μJ/pulse) in a single band (780 nm) was irradiated through the skull to the intracerebral tumor, 24 and 48 hours after LP-iDOPE injection. NIR light (Ushio Opto Semiconductors, Inc. Tokyo, Japan) was generated by a light-emitting diode (LED) with an output of 100 mW/cm^2^ for 10 minutes per tretament. Three treatments were utilized; (a) no treatment control (group A, *n* = 3), (b) LP-iDOPE injection alone (group B, *n* = 4), and (c) LP-iDOPE injection combined with LED illumination (group C, *n* = 6)). The effects of hyperthermia alone were also examined using 3 animals.

### Measurement of LP-iDOPE biodistribution

We previously reported the retention of LP-iDOPE in A549 subcutaneous tumors at 24 and 48 hours, and even at 6 days, while LP-iDOPE was completely cleared in normal tissues such as lung, liver, and kidney by 6 days [9, 10] To test the accumulation of this particle in brain tumors, LP-iDOPE was injected intravenously to 9L gliosarcoma-bearing rats and fluorescence images were obtained. Tumor-bearing rats were sacrificed 24 hours after LP-iDOPE injection to obtain fluorescence images of brain tumor. A NIR-fluorescence imaging system (IVIS lumina II, Caliper, MA, USA) was used to count photons with an XFL-HR Fluorescence Filter Option High Range (720–840 nm) detection filter.

### Magnetic resonance imaging and volumetry

To measure the volume of brain tumors, rats were examined every 7 days using a 7T magnetic resonance imaging (MRI) system (Magnet: Kobelco and JASTEC, Kobe, Japan; Console: Bruker Biospin, Ettingen, Germany) after intraperitoneal injection of 0.4 ml of Gd-DTPA (Meglumine gadpentate, Bayer, Leverkusen, Germany) 15 min before imaging. Multislice trans-axial T1-weighted MR images covering the entire brain (T1WI; multi-slice spin echo, TR/TE = 400/9.57 ms, 256 × 256, field of view = 25.6 × 25.6 mm^2^, average = 4) were acquired. In order to measure the tumor volume, MRI images were analyzed by OsiriX.

### Interleukin-2 gene transduced cell preparation

The method for IL-2 cDNA transduction was described elsewhere [30]. Briefly, a retrovirus vector LXSN containing human IL-2 cDNA was transfected into Ψ2 cells using a lipofectin reagent (Life Technologies, Inc., Gaithersburg, MD, USA). After drug selection with G418 (Life Technologies, Inc.), the culture supernatants containing retrovirus were incubated with PA317 cells in the presence of 8 μg/ml Polybrene (Aldrich, Milwaukee, WI, USA) for infection. The culture supernatants from the G418-resistant PA317 cells was used for infecting 9L cells. We then selected each clone secreting the largest amount of IL-2 (9L/IL-2). The amount of released IL-2 from 9L/IL-2 was 0.9 ng/ml/5 × 10^5^ cells/24 h.

### Histopathological analysis

After anesthetization with 2.0% isoflurane, tumor-bearing rats were perfused through the ascending aorta with 4% paraformaldehyde and brains were removed to generate formalin-fixed paraffin-embedded tissues. The paraffin-embedded samples were cut into 4 μm sections and mounted on microscopic slides. Hematoxylin and eosin (HE) staining and immunohistochemical analyses were performed. The primary antibodies used for immunohistochemistry were anti-CD8 antibody (1:200 dilution; OX-8, Serotec, Oxford, UK), anti-rat macrophage/microglia (1:100 dilution; RM-4, TransGenic Inco., Kobe, Japan), and anti-heat shock protein-70 (HSP70) (1:200 dilution; W27, Santa Cruz Biotechnology, Inc., Santa Cruz, CA, USA). TUNEL staining was performed using *In Situ* Cell Death Detection kit, POD (Roche Holding AG, Basel, Switzerland) after LP-iDOPE injection with and without NIR irradiation.

### Heating of tumor tissue

To measure the effects of heating alone on tumor tissues, a micro-heater needle (AD13025; Admetec, Ehime, Japan) was inserted into the tumor center using the stereotactic apparatus. The heating machine can precisely maintain the tissues immediately surrounding the probe at any temperature.

### Statistical evaluation

We evaluated survival time, tumor volume, and the accumulation of LP-iDOPE. To compare the survival time, we divided the glioma-bearing rats in three groups. Analysis of survival was conducted by a log-rank test based on the Kaplan-Meier method.

We analyzed MRI images to measure intracranial tumor volume. A gadolinium-based contrast media was injected to the rats intraperitoneally (1.0 ml/kg) before MRI. The OsiriX region-growing algorithm was used to measure tumor volumes. The tumor volumes were compared among the three groups using Mann–Whitney's *U*-test. These statistical comparisons were performed using Stat-View software and SAS software (SAS Institute Inc., Cary, NC, USA).
